# Minimally Invasive Delivery of Microbeads with Encapsulated, Viable and Quiescent Neural Stem Cells to the Adult Subventricular Zone

**DOI:** 10.1038/s41598-019-54167-1

**Published:** 2019-11-28

**Authors:** Rita Matta, Seyoung Lee, Nafiisha Genet, Karen K. Hirschi, Jean-Leon Thomas, Anjelica L. Gonzalez

**Affiliations:** 1 Department of Biomedical Engineering, Yale University School of Medicine, New Haven, CT 06511 United States; 2 Department of Neurology, Yale University School of Medicine, New Haven, CT 06511 United States; 3 Yale Stem Cell Center, Yale University School of Medicine, New Haven, CT 06511 United States; 4 Yale Cardiovascular Research Center, Yale University School of Medicine, New Haven, CT 06511 United States; 5 Sorbonne Universités, UPMC Université Paris 06, Institut National de la Santé et de la Recherche Médicale U1127, Centre National de la Recherche Scientifique, AP-HP, Institut du Cerveau et de la Moelle Epinière, Hôpital Pitié-Salpêtrière, Paris, France

**Keywords:** Cell delivery, Stem-cell niche

## Abstract

Stem cell therapies demonstrate promising results as treatment for neurological disease and injury, owing to their innate ability to enhance endogenous neural tissue repair and promote functional recovery. However, delivery of undifferentiated and viable neuronal stem cells requires an engineered delivery system that promotes integration of transplanted cells into the inflamed and cytotoxic region of damaged tissue. Within the brain, endothelial cells (EC) of the subventricular zone play a critical role in neural stem cell (NSC) maintenance, quiescence and survival. Therefore, here, we describe the use of polyethylene glycol microbeads for the coincident delivery of EC and NSC as a means of enhancing appropriate NSC quiescence and survival during transplantation into the mouse brain. We demonstrate that EC and NSC co-encapsulation maintained NSC quiescence, enhanced NSC viability, and facilitated NSC extravasation *in vitro*, as compared to NSC encapsulated alone. In addition, co-encapsulated cells delivered to an *in vivo* non-injury model reduced inflammatory response compared to freely injected NSC. These results suggest the strong potential of a biomimetic engineered niche for NSC delivery into the brain following neurological injury.

## Introduction

The subventricular zone (SVZ) is one of the two germinal regions in the adult mouse brain where neurogenesis occurs. The SVZ is prominent on the lateral wall of lateral ventricles (LVs), and is characterized by a highly organized and vascularized microenvironment^1,2^. Neural stem cells (NSC) interact with endothelial cells (EC) and vascular pericytes (PCs) that line the wall of infiltrating blood vessels and deliver blood borne and secreted factors^3,4^. NSC interactions with the vasculature and LVs are responsible for maintaining brain homeostasis and facilitating appropriate response to injury, particularly in the context of neurogenesis following stroke. It is well-established that the interactions between NSC and EC regulate NSC proliferation and quiescence through ephrin-B/EphB signaling^5^ and Notch signaling^6^, respectively. Under normal homeostatic condition, EC prohibit NSC differentiation and promote a quiescent state^4^. However, following neurovascular injury, such as stroke, EC junctions and extracellular matrix (ECM) are disturbed by oxygen and glucose deprivation resulting from the loss of cerebral blood flow. Consequently, damaged EC are unable to regulate typical NSC function, resulting in enhanced NSC proliferation and neuronal differentiation^7^. Despite the activation of NSC, endogenous repair mechanisms are insufficient to foster recovery and functional replacement of damaged neurons.

Stem cell therapies are currently considered a viable opportunity for replacement of lost neural populations and restoration of neural circuitry following injury. However, current therapies require improved engraftment strategies for better cell survival, which in turn can help expedite and enhance the generation of new cells in the cytotoxic area following cerebral ischemia^8,9^. To address these limitations, tissue engineering advances provide a promising platform to facilitate the recovery of damaged tissue^10^. Recently, hydrogel scaffolds were designed to enhance stem cell delivery, taking into consideration the biocompatibility, biodegradation, and mechanical stresses associated with integration of cells to a hydrogel^11–14^. As demonstrated by these efforts, effective tissue engineered approaches to improve behavioral and functional recovery must promote exogenous cell survival and transplantation, while promoting endogenous repair mechanisms.

Here, we have created a polyethylene glycol (PEG)-based scaffold for NSC and EC encapsulation and delivery into the mouse SVZ for cell engraftment into host tissue. PEG, known for its bioinert nature, can be chemically modified by immobilizing biologically active peptide sequences from the ECM to promote cell interactions *ex viv*o, thereby supporting cell adhesion among other cell functions^15–17^. The tunable nature of polymeric platforms facilitates the engagement of multiple cell types. Two of the most abundant ECM proteins in neural tissue are fibronectin and laminin^9^, where the fibronectin derived peptide arginine–glycine–aspartic acid-serine (RGDS) and laminin derived peptide tyrosine-isoleucine-glycine-serine-arginine (YIGSR) have each demonstrated the ability to support cell adhesion of EC and NSC, respectively^15,18^. In this study, we have conjugated these adhesive peptides to PEG at high efficiency to promote dual-cell adhesion. To facilitate degradation of PEG *in vivo*, we have also conjugated the degradable sequence glycine-glycine-leucine-glycine-proline-alanine-glycine-glycine-lysine (GGLGPAGGK; LGPA) which is susceptible to degradation by cell-secreted collagenases^18,19^, allowing for the extravasation of encapsulated cells and promoting exogenous cell integration within host tissue.

In the present study, we have created and optimized the generation of cell-encapsulated PEG microbeads with PEG-YIGSR and PEG-RGDS to support both mono- and co-culture systems of NSC and EC. Importantly, we demonstrate that EC enhance NSC survival and maintain NSC quiescence within our co-culture system, *in vitro*. Furthermore, EC accelerate the degradation of PEG microbeads through proteolytic MMP activity, facilitating the escape of NSC from the microbeads. Consistent with our *in vitro* results, we found that co-encapsulated NSC and EC delivered to the murine SVZ in the microbeads retained NSC quiescence and accelerated microbead degradation. Moreover, cells encapsulated within microbeads diminished immune cell infiltration and increased the number of endogenous SVZ neural stem and progenitor cells, following their delivery *in vivo*, as compared to freely injected cells. Our work provides strong evidence that engineered PEG-based mimics of the neurovascular niche may serve as a valuable delivery device for cell therapies in response to neurological disease.

## Results

### Addition of 0.25% pluronic is optimal for microbead diameter constraint and encapsulated cells

**Figure 1:**
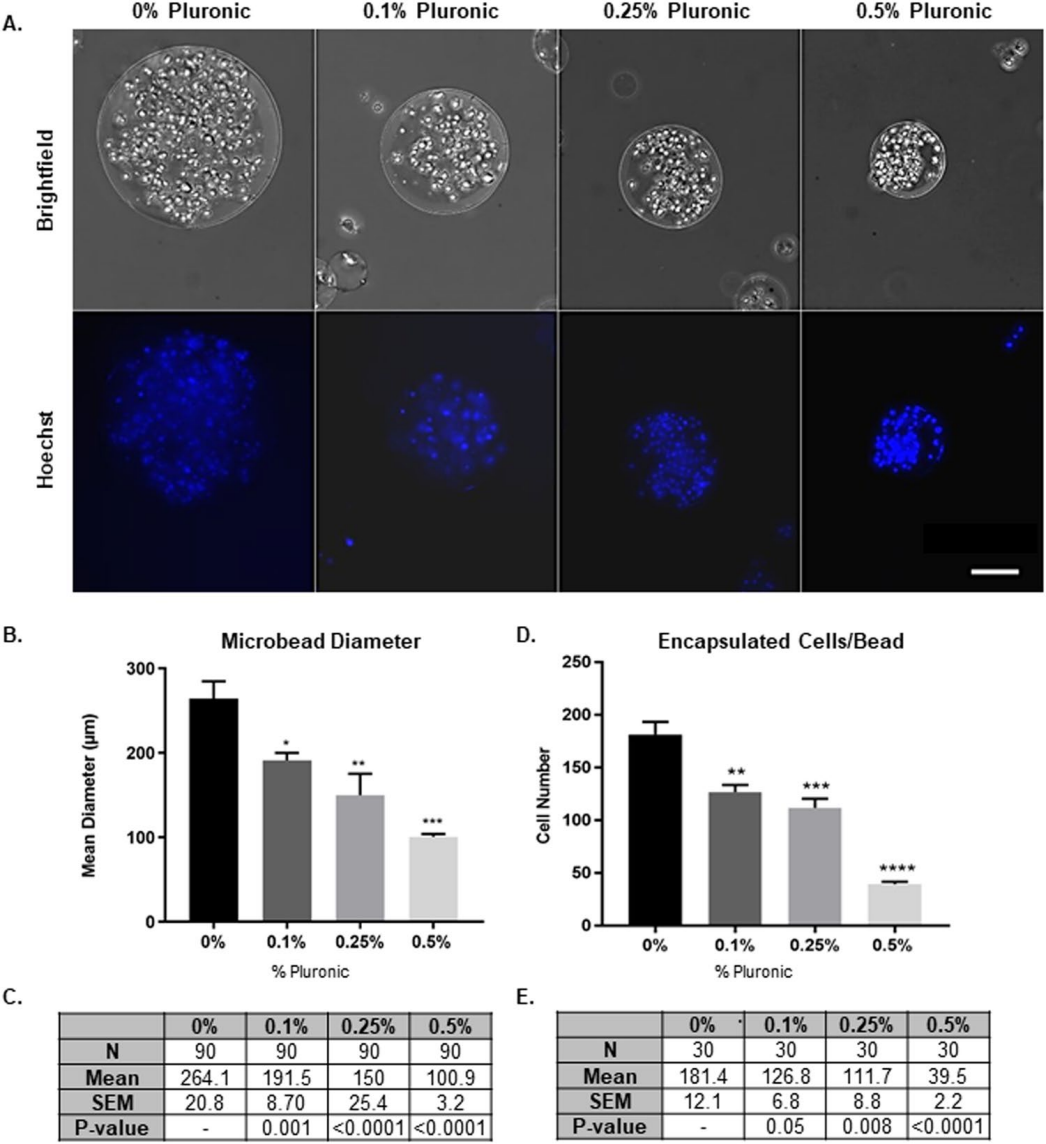
Creation and Optimization of Cell-Encapsulated Microbeads. (**A**) Brightfield images of NSC encapsulated in microbeads (top row) and nuclei stained with Hoechst (bottom row), demonstrating influence of Pluronic concentrations from 0 to 0.5% additions. (**B**) Microbead mean diameter for varying Pluronic concentration (n = 3). Values tabled in (**C**). (**D**) Cells encapsulated per bead for varying Pluronic concentrations (n = 3). Values tabled in E. Error bars represent SEM. Scale bar (50 µm) representative of all images. *p ≤ 0.05, **p ≤ 0.01, p ≤ 0.001, and ****p ≤ 0.0001 compared to 0% Pluronic determined by 1way ANOVA with multiple comparisons.

Using an oil emulsion technique, we produced microbeads with a mean diameter of 100–150 μm while maximizing the number of encapsulated cells. Using polymer and photochemicals in an oil emulsion, we obtained a microbead diameter of 264.1 ± 20.81 μm with 181.4 ± 12.1 cells encapsulated per bead. To achieve microbeads of our target size with encapsulated cells, we incorporated Pluronic, a PEG-based and cell compatible surfactant, at various concentrations: 0.1, 0.25, and 0.5% (v/v) (Fig. 1A), yielding a mean diameter of 191.5 ± 8.70 μm, 150 ± 25.38 μm, and 100.9 ± 3.23 μm, respectively (Fig. 1A). With increased concentration of Pluronic, the mean bead diameter decreases significantly (Fig. 1C). Using a comparable starting cell concentration, 0.1, 0.25, and 0.5% (v/v) concentrations of Pluronic facilitated the encapsulation of 126.8 ± 6.79, 111.7 ± 8.76, and 39.5 ± 2.17 cells, respectively (Fig. 1D,E). A 0.25% Pluronic addition was chosen for *in vitro* and *in vivo* experimentation in subsequent studies, yielding microbeads with a mean diameter of ~150 μm, containing ~112 cells encapsulated per bead, thereby meeting our size constraint for adequate oxygen delivery to the core of the microbead.

### Co-encapsulation of EC and NSC promotes NSC quiescence and enhances NSC viability

**Figure 2:**
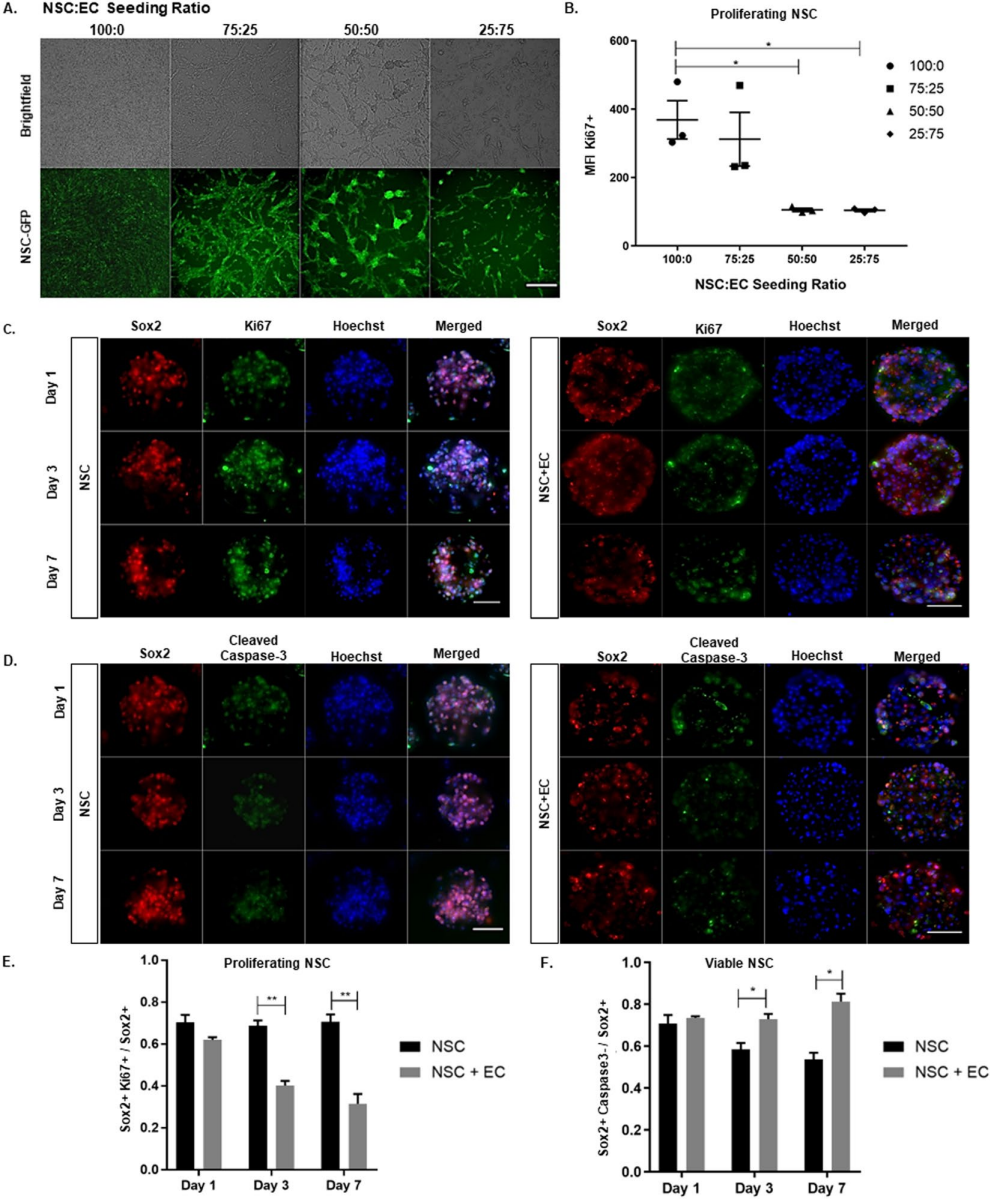
Co-encapsulation of EC and NSC Promotes NSC Quiescence and Enhances NSC Viability. NSC:EC seeding density depicting brightfield (top row) and GFP-tagged NSC (bottom row) at 4 different ratios (**B**) Quantification for cell mean fluorescence intensity for Ki67^+^ NSC 4 different ratios of NSC:EC determined through flow cytometry (n = 3). (**C**) Fluorescent images of NSC mono- and co-culture (left and right panel, respectively), stained for Sox2, Ki67, Hoechst, and merged. Imaged at 1, 3, and 7 days (top, middle, bottom panel, respectively) with quantified Sox2 + Ki67+ cells in E (n = 3). (**D**) Fluorescent images of NSC mono- and co-culture stained for cleaved caspase-3 with quantified Sox2+ Caspase3- cells in F (n = 3). Error bars represent SEM. Scale bar (50 µm) representative of all images. *p ≤ 0.05, ***p ≤ 0.001, ****p ≤ 0.0001 determined by an unpaired t test.

Delivery of NSC in an undifferentiated, quiescent state is critical for NSC to respond properly to injury. EC induce NSC quiescence through Notch signaling as an effect of cell-to-cell contact^20^. In order to determine the optimal ratio of NSC:EC for co-encapsulation, a seeding curve was conducted for 4 different ratios: 100:0, 75:25, 50:50, and 25:75, distinguishing NSC from EC in live culture by using GFP transfected NSC (Fig. 2A). Mean fluorescence intensity (MFI) of Ki67^+^ NSC was determined through flow cytometry (Fig. 2B). NSC:EC ratios of 100:0 and 75:25 produce a MFI of 368.7 ± 55.97 and 312 ± 78.5, respectively. However, a 50:50 seeding ratio significantly reduces MFI to 105.4 ± 5.0. In this way, Ki67^+^ NSC are reduced approximately 3-fold. Although a seeding ratio of 25:75 results in the greatest reduction of proliferating NSC, there is not a significant difference in MFI between the 50:50 and 25:75 seeding densities. In addition, a seeding density of 25:75 would diminish the effective goal of delivering an abundance of NSC, as a 25:75 ratio construct would be majority EC. From this point on, studies were conducted at a 50:50 encapsulation ratio of NSC:EC.

Once our encapsulation density was optimized, we evaluated NSC proliferation via immunostaining for NSC marker Sox2 and proliferative marker Ki67 (Fig. 2C). For mono- and co- encapsulated microbeads, we quantify proliferating NSC as Sox2^+^Ki67^+^ cells, normalized to the total number of Sox2^+^ cells (Fig. 2E). One day following encapsulation, we observed a Sox2^+^Ki67^+^/Sox2^+^ ratio of 70.41 ± 3.57 and 62.07 ± 1.19 proliferating NSC in the mono-and co-culture system, respectively. Importantly, by day 3, we observed a reduction of Ki67^+^ NSC in the co-culture to 40.23 ± 2.19 compared to NSC alone, with a proliferating population of 68.76 ± 2.52. This significant reduction of proliferating NSC in the co-culture is maintained by day 7, where the mono-and co-culture present 70.72 ± 3.49 and 31.53 ± 4.65, respectively, suggesting that NSC maintain a non-proliferative, quiescent state in the presence of EC.

Another major limitation to successful stem cell delivery is survival of transplanted cells in the cytotoxic and inflamed brain tissue^21^. To probe NSC viability in mono- and co- encapsulated microbeads, we stained for apoptotic marker cleaved caspase-3 (Fig. 2E), quantifying Sox2^+^Caspase-3^+^ cells normalized to Sox2^+^ cells in order to assess NSC viability (Fig. 2F). One day following encapsulation, the ratio of cell viability in mono and co-cultures was 70.73 ± 4.18 and 73.50 ± 0.81, respectively. However, by day 3, NSC encapsulated alone had a significant decrease in viability to 58.51 ± 3.01 compared to the co-culture viability of 72.92 ± 2.48. Similarly, at day 7, NSC alone presented a significant decrease in viability to 53.62 ± 3.25 compared to the co-culture viability of 81.27 ± 3.75, suggesting EC play a role in promoting NSC viability.

### EC MMP activity facilitates degradation of microbeads

**Figure 3:**
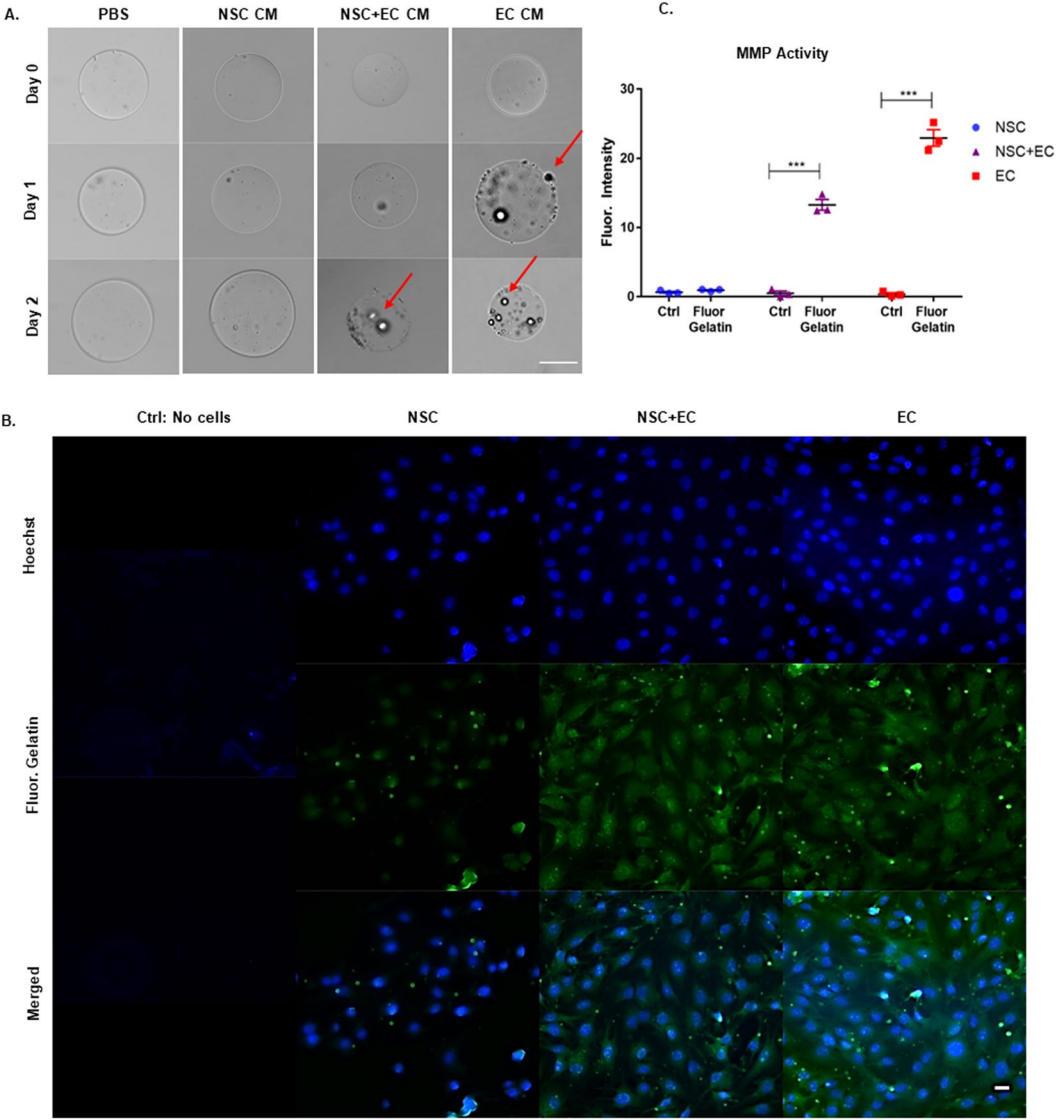
EC MMP Activity Facilitates Degradation of Microbeads. (**A**) Degradable microbeads in PBS, collagenase, NSC culture media (CM), NSC + EC CM, and EC CM, at 0, 1, and 2 days. Arrows point to areas of microbead surface corruption. (**B**) *In situ* zymography with no cells (control), NSC, NSC and EC and EC monolayers. Cells stained with Hoechst (top row) are incorporated into an agarose gel with fluorescein gelatin to detect cell-secreted MMPs (middle row), quantified in (**C**) using ImageJ (n = 3). Error bars represent SEM. Scale bar (50 µm) representative of all images. **p ≤ 0.01 determined by an unpaired t test.

Cell extravasation from the scaffold and integration into the host tissue is necessary post-delivery. The incorporation of the collagenase-sensitive peptide LGPA renders microbeads specifically susceptible to degradation by cell-secreted MMPs. The LGPA peptide has a cleavage site between leucine and glycine, demonstrating specificity only to collagenase degradation^22,23^. To assess the effect of cultured media (CM) on degradable microbeads, CM of either NSC, NSC and EC, or EC were added to degradable microbeads (Fig. 3A). Changes in microbead morphology and integrity with NSC CM resembled microbeads in PBS, demonstrating no change in shape of the beads, however that of EC CM demonstrated a bead hallowing effect and surface erosion within 24 hours. NSC and EC CM began to promote microbead morphological changes within 48 hours. (Fig. 3A). This suggests CM with EC-secreted MMPs accelerated microbead degradation and that the use of a co-encapsulated cell construct would be beneficial for cells to escape the beads at a faster rate than NSC encapsulated alone.

To further assess MMP activity, *in situ* zymography was performed using a fluorescein salt conjugated to gelatin, where gelatin serves as a target for collagenases. MMP-2 and −9 degradation^24^. Quantification of cell enzymatic activity was determined through the mean fluorescence intensity normalized to cell number, subtracting background. The gelatin substrate containing quenched fluorogenic molecule demonstrated a robust and significant proteolytic activity of EC (22.93 ± 1.18) compared to NSC (0.92 ± 0.10) (Fig. 3B,C). Interestingly, NSC with EC demonstrated proteolytic activity in a linear fashion compared to the two cell types alone (13.26 ± 0.77), suggesting EC are the major contributor to MMP activity in the co-culture condition. These data suggest that EC are necessary to facilitate NSC extravasation from the engineered microbead and into the host tissue.

### EC accelerate quiescent NSC extravasation from microbead *in Vivo*

**Figure 4:**
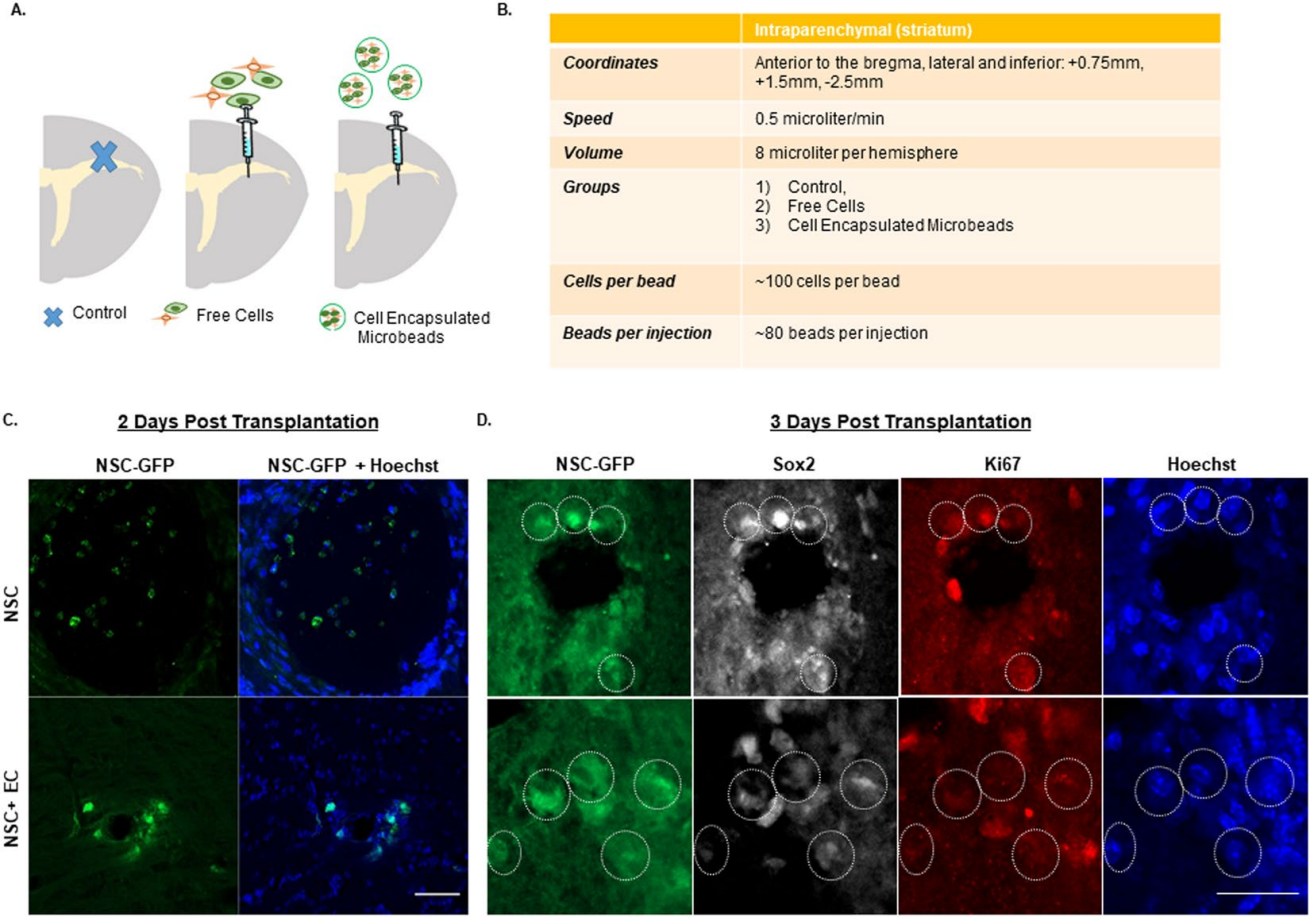
EC Accelerate Quiescent NSC Extravasation from Microbead. (**A**) Schematic overview depicting the transplantation target site (striatum) in the adult mouse brain for control, free cells, and cell encapsulated microbeads with transplantation parameters outlined in (**B**). (**C**) Confocal images of GFP-tagged NSC and Hoechst mono- and co-cultures encapsulated in microbeads to observe cell extravasation at 2 days post transplantation and (**D**) expression of GFP-tagged NSC, Sox2, Ki67, and Hoechst to observe cell proliferation at 3 days post transplantation. Scale bar (25 µm) representative of all images.

The transplantation strategy of microbeads is depicted in Fig. 4A with injection parameters outlined in Fig. 4B. A needle stab injury was used to cause direct injury to the cortex, corpus callosum and striatum. This intracerebral injection induced an alignment of Iba1^+^ microglial cells and GFAP^+^ astrocytes along the needle tract (Supplementary Fig. 2). For delivery of the NSC, encapsulated mono-, co-cultures, or freely injected cells were prepared on the day of implantation. A total of ~1 × 10^4^ cells encapsulated in microbeads were stereotaxically transplanted into the striatum (coordinates: 0.75 mm anterior and 1.5 mm lateral to the bregma, and 2.5 mm ventral) of adult male mice (8–10 weeks old). In order to observe the response of microglia and tissue macrophages to grafted encapsulated cells, no immunosuppressive drug treatment was administered to mice prior to injection. Importantly, when we analyzed the brain slices at day 2, we found that the microbeads encapsulated with co-cultures were completely degraded, allowing for NSC to migrate into the surrounding tissue (Fig. 4C). Notably, the microbeads loaded with monoculture remained intact, supporting the observation that EC expedite microbead degradation (Fig. 3). Additionally, consistent with our *in vitro* results (see Fig. 2C,D), delivered NSC remained quiescent (GFP^+^/Sox2^+^/Ki67^−^) when encapsulated with EC, whereas those encapsulated alone were increasingly dividing/proliferating (GFP^+^/Sox2^+^/Ki67^+^; Fig. 4D).

### Transplanted NSC in microbeads increase endogenous NSC proliferation and differentiation in SVZ

**Figure 5:**
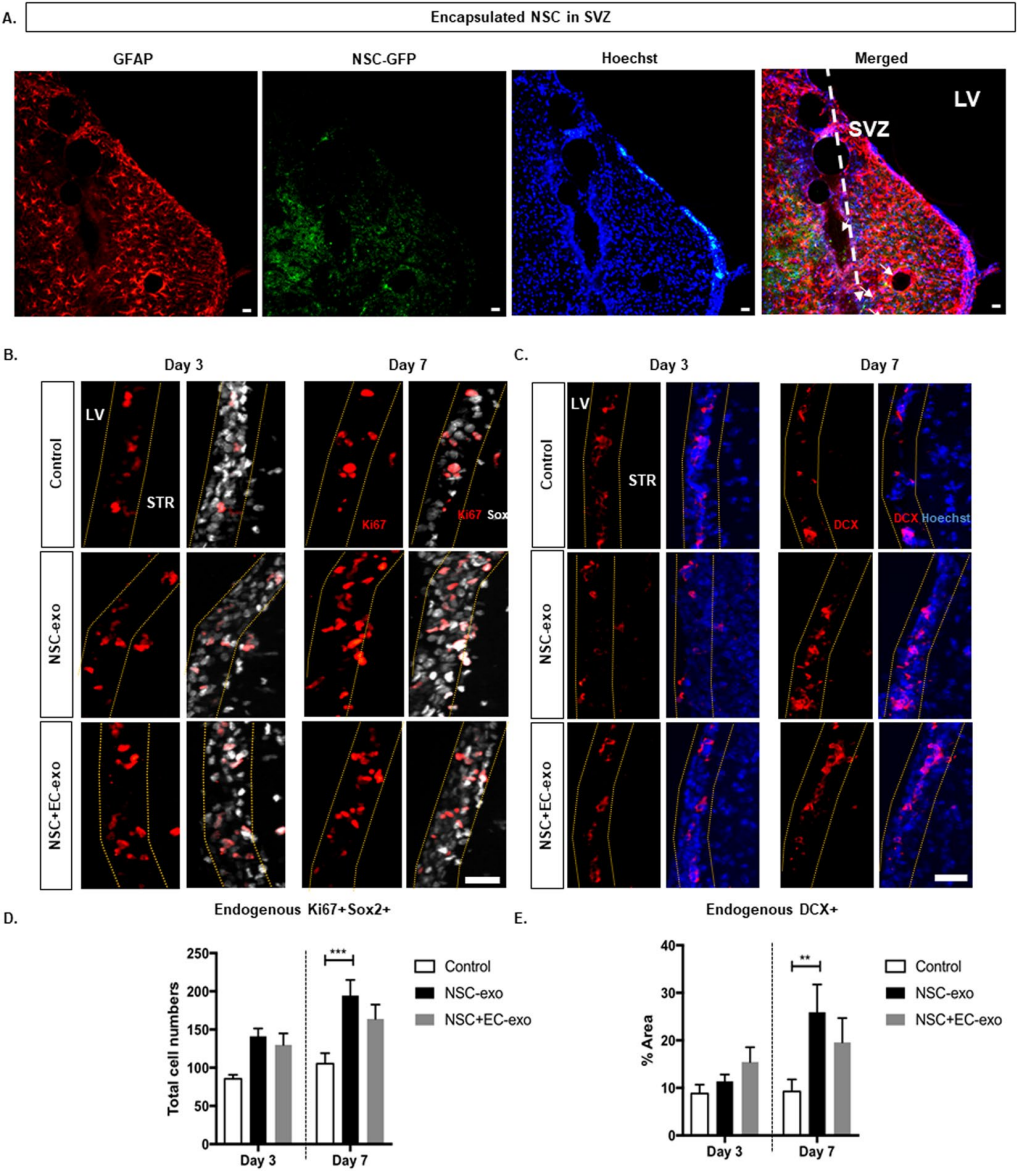
Encapsulated NSC  Increase Endogenous Neural Stem Progenitor Cell Proliferation and Differentiation. (**A**) Confocal image of injected microbeads position into the striatum relatively to the lateral ventricle (LV). Few GFP-tagged NSC were observed migrating towards SVZ two weeks after injection, however at day 3, no GFP-tagged NSC were founded in the SVZ. (**B**) Confocal images staining for Ki67 and Sox2 stained tissue sections of control (upper panel), encapsulated NSC (middle panel) and encapsulated NSC and EC (lower panel) groups, quantifying co-localization of proliferating NSC (Ki67^+^/Sox2^+^) in SVZ tabled in (**D**). (**C**) Confocal images staining for DCX and Hoechst to visualize endogenous NSC maturation in SVZ, quantifying endogenous DCX^+^ cells tabled in E. Error bars represent SEM. Three different SVZ regions were selected from each brain tissue for the analyses (n = 3-5). **p < 0.01 determined by one-way ANOVA with multiple comparisons. Scale bar (25 µm) representative of all images.

Microbeads were delivered into the striatum, at a distance which is 250 μm ± 150 μm from the SVZ, at most, 50–100 μm to the lateral ventricle wall. At one week after implantation, encapsulated GFP-tagged NSC remained within the local vicinity of microbeads. Due to their limited migration, we do not see mixing of engrafted GFP-tagged NSC populations with endogenous SVZ located NSC (Fig. 5A). To determine whether exogenous NSC can affect the endogenous NSC activity in the SVZ, brain tissue sections were prepared at 3 and 7 days post-transplantation (dpt), and subjected to staining against Sox2, Ki67, and neuroblast marker, doublecortin (DCX). Quantitative analysis demonstrated that, at 3 dpt, the number of proliferating endogenous neural stem and progenitor cells (Ki67^+^Sox2^+^) were found to increase by 71.01 ± 6.66% and 60.57 ± 24% in mono- and co-encapsulation groups, respectively, compared to the control group (Fig. 5B). At 7 dpt, a similar increase (67.48 ± 20.1%) in the number of endogenous Sox2^+^/Ki67^+^ cells was observed for the co-culture group compared to the control group. On the other hand, a significantly higher increase (~113.8 ± 28.18%) in this cell population was observed in monoculture group compared to the control group. The percentage of endogenous DCX^+^ cells along the SVZ wall, although essentially unchanged at 3 dpt, was significantly increased (~3-fold) at 7 dpt in the mono-culture group (Fig. 5C). However, this cell population remained unchanged in co-culture group from 3 to 7 dpt. These results indicate that the exogenous NSC released from transplanted microbeads promote endogenous neural stem and progenitor cell proliferation and differentiation in the SVZ. On the contrary, exogenously delivered EC had a subtle but potent effect on endogenous neural stem and progenitor cell activity to inhibit their differentiation into newborn neurons.

### Co-encapsulation of EC and NSC Ameliorates Leukocyte Infiltration, Microglial Activation and Cell Death at the Site of Injection

**Figure 6:**
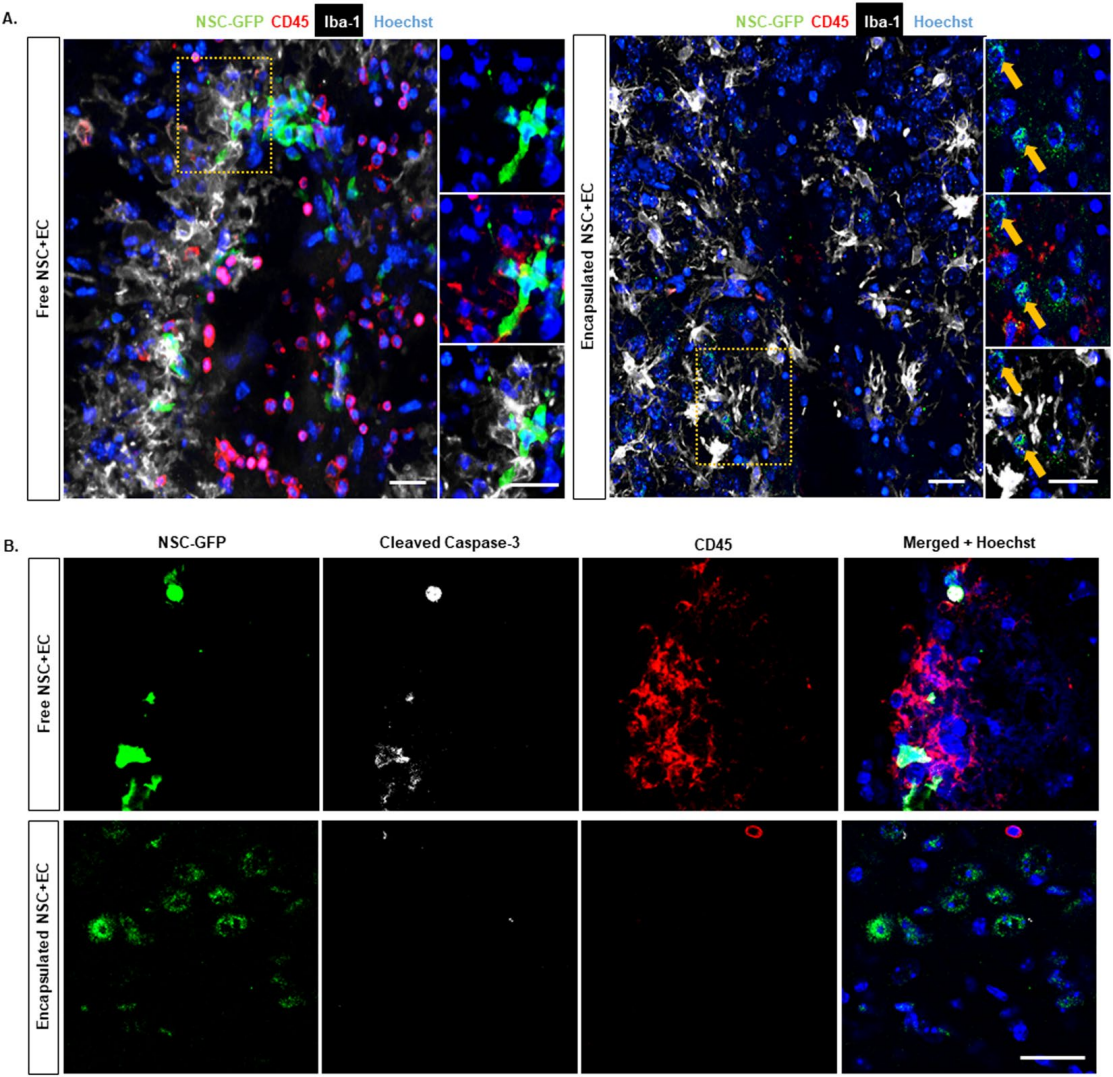
Encapsulated Cells Exhibit Less Immune Response Compared to Free Cells. (**A**) Immunofluorescence representative images of free (left panel) and encapsulated (right panel) GFP-tagged NSC with EC at 2 days showing expression of GFP-tagged NSC, CD45, Iba-1, and Hoechst. (**B**) Confocal microscopy showing colocalization of anti-GFP, cleaved caspase-3, CD45 and Hoechst in free (top panel) and encapsulated (lower panel) co-culture transplantation groups. Scale bar (25 µm) representative of all images.

The local immune response to transplanted exogenous NSC and its modulation are critical in enabling successful exogenous cell engraftment. Previous studies have indicated promising characteristics of PEG-based microbeads for producing minimal inflammation when transplanted into brain tissue^25^. To evaluate the local inflammatory response to the implanted microbeads, the extent of leukocyte (CD45) recruitment and microglia (Iba1^+^/CD45^−^) response were compared between encapsulated co-culture and control (freely injected co-culture) groups (Supplementary Fig. 2). As shown in Fig. 6, free co-cultures induced a greater leukocyte infiltration around the injection site compared to the encapsulated co-cultures 2 days after transplantation (Fig. 6A,B). Iba1 + microglia were found in close proximity of the injection site in both groups (Fig. 6A). Interestingly, while most, if not all, NSC (GFP^+^) were colocalized to Iba1^+^ cells with retracted processes in the control group, they were not colocalized with Iba1^+^ cells in the encapsulated co-culture group. We next examined whether microbead encapsulation protects exogenous NSC from apoptotic cell death by immunohistochemistry for cleaved caspase-3 in both groups. The viability of NSC + EC that were freely injected was 33.37% ± 16.74. In contrast, there was a significant increase (*p* value = 0.0239) in the viability of NSC that were encapsulated and delivered with EC at 2 days following transplantation (75.92% ± 6.81) (Fig. 6B).

## Discussion

A major limitation to cell based neurorestorative therapies is the engraftment of undifferentiated, viable cells to host tissue. In particular, NSC^26^ and bone marrow stromal cells^27,28^ delivered directly to stroke injury models have shown a fraction of engrafted cells undergoing differentiation into mature neurons post injection, however the *in vivo* survival rate is extremely low^9^. The use of other stem cell types, such as induced pluripotent stem cells, is limited by a lack of control over *in vivo* differentiation^29^. Another setback of stem cell injection is the extremely cytotoxic microenvironment of the post brain trauma. This injured tissue can lead to cell engulfment by infiltrating leukocytes or activated microglia. Hence, a tissue engineered scaffold that can deliver viable cells to the host tissue while maintaining a quiescent cell state presents a potential therapeutic advantage to recover the effects of neurodegeneration. The scaffold can also serve to protect encapsulated cells from immune cells that are recruited during early phases of cell transplantation, preventing exogenous cell apoptosis by ensheathing cells temporarily.

PEG, known for its tunable properties, can be used to serve as a biomimetic scaffold by which native ECM ligands for cell adhesion and spreading can be presented to cells. Here, we have utilized PEG as a scaffold for microencapsulation, mimicking the positive influence of EC on NSC in the SVZ niche. We have functionalized our scaffold, incorporating laminin-derived YIGSR and fibronectin-derived RGDS peptides to mimic two of the most abundant ECM proteins of the SVZ. By doing so, we take into account the biochemical properties seen *in vivo* to support dual-cell encapsulation. Two critical considerations when optimizing our protocol included 1) maintaining the integrity of microbeads taken up through a syringe needle for *in vivo* delivery and 2) protecting cells from diffusion-limited oxygen and glucose access upon encapsulation. Optimization of our oil emulsion through addition of 0.25% Pluronic, a cell-compatible and PEG-based surfactant, has allowed us to produce microbeads with a mean diameter of 100–150 microns, facilitating uptake through a syringe needle and allowing for diffusion to cells at the core of the scaffold. Importantly, the size of these microbeads is below the threshold of 200 μm, where hydrogels at this size may threaten the diffusion of nutrients and oxygen to the core of the scaffold. Through conjugation of MMP degradable LGPA peptide to the PEG domain, we allow cell extravasation from the microbead post-injection through a syringe needle, allowing for integration of viable, quiescent NSC in the presence of EC into the murine striatum.

The vasculature of the SVZ is a critical component of the NSC niche where direct cell-cell contact between EC and NSC enforce quiescence and promote stem cell identity. Quiescence and stem cell identity are maintained, at least in part, through EC presented ephrinB2 and Jagged1 signaling, respectively, which maintain NSC in a pro-differentiation state^20^. Recently, EC amyloid precursor protein has also demonstrated to support the maintenance of NSC quiescence^30^. In turn, NSC promote EC formation of capillary tube formation, suggesting a continued collaborative effort between NSC and EC in the SVZ niche^31,32^. Our experimental NSC:EC seeding density curve, suggested that NSC and EC encapsulation at an equal ratio (50:50) would support maintenance of NSC quiescence and survival during the transplantation process. Our results demonstrate that NSC have increased viability and maintained non-proliferative state in the presence of EC prior to and post transplantation. Finally, we demonstrate the neuroprotective effect of our PEG scaffold, protecting cells from the activated immune cells post injection, and ultimately promoting exogenous cell viability.

NSC offer a promising therapeutic benefit in restructuring and repairing cytotoxic brain tissue by replacing cells that were lost following cerebral ischemia. Although NSC are one potential source of cell for transplantation into the injured brain, there are a range of complications of delivering NSC, including the control of NSC survival, proliferation, and differentiation post-delivery^7^. To address these limitations, an increasing number of studies have investigated the potential of co-transplantation therapy to enhance NSC viability in disease models such as stroke. For instance, it was previously reported that when NSC were co-transplanted with astrocytes in a rat model of stroke, there was a higher ratio of transplanted NSC survival and proliferation, and neuronal differentiation, compared with transplanting NSC alone^33^. However, as mentioned previously, it is crucial to maintain NSC quiescence upon delivery as activated exogenous NSC may disrupt endogenous repair mechanisms. Interestingly, our *in vivo* results observing the effect of exogenous NSC on endogenous SVZ-NSC activity are in line with previous studies demonstrating that transplanted NSC from various sources, including humans^34^ and rats^35^ have the potential to induce endogenous NSC to proliferate and differentiate. Furthermore, our co-culture transplantation of NSC and EC showed that exogenous EC not only promote quiescence of exogenous NSC but also have an impact on endogenous NSC to some degree. Additional studies have reported the successful co-transplantation of freely injected EC and neural stem/progenitor cells to stroke-induced mice cortex, demonstrating survival and proliferation of neural stem/progenitor cells, in addition to accelerated neuronal differentiation^7^. Through the reduction of the localized inflammatory response observed of our encapsulated delivered niches, we predict that, in a pathological state, co-encapsulated cells will have increased viability as compared to freely injected cells.

In the present study, we focused on the role of EC in promoting NSC survival and quiescence, ignoring to date the potential impact of other cells native to the SVZ, including ependymal cells, pericytes, and microglia. Through the use of our tunable PEG system, we can continue to optimize the microbead environment to include additional adhesive and degradative ligands to accommodate additional cell types and facilitate additional cell-cell and cell-ECM interactions. In doing so, we will create an SVZ *ex vivo* biomimetic niche for optimized delivery of healthy and viable reparative cells. Our *in vivo* data indicate promising results in a non-injury murine model, and future work will transition the use of encapsulated neural stem and endothelial cells to a stroke injury model. We can then observe the integration of cells in the inflamed, large cavity, as we continue to optimize our microbead formulation to protect cells post transplantation with the ultimate goal of promoting functional and long-term recovery.

## Experimental Procedures

All experiments were performed in accordance with relevant guidelines and regulations.

### Cell maintenance

The features of this cell line have been characterized to establish growth of NSC as pure culture in defined serum-free conditions. In brief, established adherent neural cell lines (ANS4; kindly provided by S. Pollard) were cultured in serum-free basal medium supplemented with N2 and B27 1 mg/mL laminin, and 10 ng/mL EGF and FGF-2 growth factors. For tracking *in vivo*, GFP transfected ANS4s were utilized^36^. The commercially available immortalized mouse brain endothelial cell line (bEND.3, ATCC) was cultured according to manufacturer’s protocol. In brief, cells were cultivated in DMEM with 4 mM L-glutamine, 4500 mg/L glucose, 1 mM sodium pyruvate, and 1500 mg/L sodium bicarbonate, 10% fetal bovine serum and 1× Penicillin/Streptomycin. For both NSC and bEND.3 cells, culture medium was changed three times weekly at a seeding density of 2–2.5 × 10^5^ cells/T25 flask and 4 × 10^6^ cells/T75 flask, respectively. Cells were maintained at 37 °C in 5% CO_2_ atmosphere and passages 27–33 were used for all studies.

### Synthesis of polymer and microbeads

#### PEGDA synthesis

Poly (ethylene glycol) diacrylate (PEGDA) was prepared as previously described^15,16^. Briefly, dry 10,000 Da PEG (0.1 mmol/ml) was combined with acryloyl chloride (0.4 mmol/mL) and trimethylamine (0.2 mmol/mL) in anhydrous dichloromethane, to react in argon overnight. The solution reacts with potassium carbonate to allow for phases to separate. Lastly, the lowest organic phase is dried with magnesium sulfate and precipitated with diethyl ether, filtered, and dried overnight.

#### Bioactive and degradable polymer synthesis

The peptide sequences utilized for cell adhesion and degradation properties were RGDS, YIGSR, and LGPA. The LGPA was synthesized with > 85% purity and characterized by free HPLC and mass spectroscopy. Bioactive peptides were conjugated to PEG as previously described^15,16^. RGDS and YIGSR were conjugated to PEG monoacrylate by reacting the peptides respectively with acryloyl-PEG-N-hydroxysuccinimide Ester (PEG-NHS, 3500 Da) in 50 mM sodium bicarbonate (pH 8.5) at a 1:1 (peptide:PEG) molar ratio for 2 hours at room temperature. PEGylated products were then dialyzed for 24 hours using SnakeSkin™ Dialysis Tubing, frozen, and lyophilized. NMR was conducted to observe the disappearance of the proton peak of NHS at 2.9 mM. The collagenase degradable LGPA sequence was conjugated to PEG as previously described^22^, reacting the peptide in sodium bicarbonate at a 1:2 (peptide:PEG) molar ratio for 24 hours at room temperature. PEG-LGPA-PEG was then dialyzed, frozen, and lyophilized.

#### Microbead synthesis

Microbeads are formulated based on an oil emulsion technique previously described^37,38^ which have been optimized to create non-degradable, fluorescent microbeads in addition to degradable or non-degradable microbeads with encapsulated NSC +/− EC. Our methods have been aimed to increase packing density into a 24 G Hamilton syringe without damaging the integrity of the microbeads, as well as maximize the number of NSC and EC encapsulated. To meet this criteria, we incorporated Pluronic, a PEG-based and cell compatible surfactant, at various concentrations: 0.1, 0.25, and 0.5% (v/v), determining the optimal addition to be 0.25% used in subsequent *in vitro* and *in vivo* studies.

Cell-Encapsulated Microbeads: An aqueous pre-gel solution was prepared with the additions of 0.1 g/mL 10 kDa PEGDA, 1.5% (v/v) triethanolamine in 1X Dulbecco’s Phosphate Buffered Saline (PBS), 3.4 μl/mL 1-Vinyl-2-pyrrolidinone, 10 μM Eosin Y, and 0.25% (v/v) Pluronic. Photoinitiator (300 μg 2, 2-dimethoxy-2-phenyl-acetophenone/mL NVP) was added at a concentration of 3 μL/mL of mineral oil in a glass tube.

Due to PEG being an inert polymer, we incorporated fibronectin-derived peptide RGDS and laminin-derived peptide YIGSR to render the polymer bioadhesive. Concentration of adhesive peptides were determined following a titration curve (2–6 mM) by seeding cells on coverslips coated with polymer in PBS to ensure the concentration was sufficient for cell attachment (not shown). The concentration of photochemicals and the methodology remains consistent as described above. For mono-cultures of NSC, 4 mM PEG-YIGSR was added, where for co-cultures 2 mM PEG-YIGSR and PEG-RGDS were added. To encapsulate cells, NSC and EC were lifted using Accutase or 0.25% Trypsin/EDTA, respectively, and resuspended in pre-gel at a final concentration of 75 × 10^6^ cells/ml. For co-culture encapsulation, a 50:50 ratio of NSC:EC was used and cultured in NSC media. Photoinitiator (300 μg 2, 2-dimethoxy-2-phenyl-acetophenone/mL NVP) was added at a concentration of 3 μL/mL of mineral oil in a glass tube. To the mineral oil and photoinitiator phase, 25 μl pre-gel and cell solution was added, and the phases were vortexed for 13 seconds. The oil emulsion phase was then exposed to white light from a fiber optic illuminator (Cole Parmer High Intensity Illuminator, 67% intensity) for 3 minutes. Microbead were separated from the oil phase by adding 1 ml of NSC media and centrifuging for 5 minutes at 1200 RPM, decanting between three washes. Microbeads were transferred to a multiwell plate for culture in NSC media.

Verification of Microbead Synthesis Parameters Prior to Cell Encapsulation: A titration assay was done for all photochemicals mentioned above, adding the pre-gel solution to a monolayer of NSC and EC (not shown here). Final concentrations chosen were non-toxic, where cells maintained their morphology in this aqueous solution. Monolayers of cells were exposed to white light as to mimic the photo-crosslinking process, and similarly, cells maintained their morphology. Similar mechanical agitation was done in pre-gel solution in the absence of white light (emulsifying in mineral oil, vortexing, and centrifuging/decanting the mineral oil several times). Once cells were collected from the separated phases and seeded in media, they were healthy. This suggests the chemical and mechanical aspects of our protocol are non-toxic and non-damaging to cell integrity.

Degradable, Cell-Encapsulated Microbeads: For *in vivo* studies with encapsulated cells, 0.1 g/mL 10 kDa PEGDA was replaced with 0.1 g/mL PEG-LGPA-PEG to allow cells to extravagate from the microbeads and integrate with host tissue. Degradation assessment was conducted using 2 mg/ml collagenase, artificial cerebrospinal fluid (CSF), and media (control) to observe complete degradation within 24 hours in collagenase buffer (Supplemental Fig. 1).

### Characterization of cell response in mono and co-culture

#### NSC:EC Seeding Ratio

GFP transfected NSC and EC were seeded in 12 well culture plates at a final seeding density of 0.1 × 10^6^ cells. To determine the optimal NSC:EC seeding ratio, a seeding density curve was conducted at 100:0, 75:25, 50:50, and 25:75 cells. After one day, wells were imaged, distinguishing NSC from EC via GFP tag expression. NSC were lifted using Accutase, which does not lift EC, ensuring EC remained in the well post NSC collection. NSC were then fixed in 4% PFA for 10 minutes, blocked and permeabilized with 5% normal donkey serum and 2% Triton X-100 in PBS for 1 hour at room temperature, and stained overnight against Ki67. Following secondary antibody incubation, cells were assessed through flow cytometry (n = 3) and quantification was conducted using FlowJo.

#### Immunocytochemistry

Cell survival and cell quiescence after 1, 3 and 7 days of culture was assessed via quantification of cell apoptotic marker cleaved caspase-3 and proliferative marker Ki67 positive immunofluorescent staining, respectively. Specifically, microbeads with encapsulated NSC + /-EC were fixed in 4% PFA for 10 minutes, rinsed, and blocked and permeabilized with 5% normal donkey serum and 2% Triton X-100 in PBS for 1 hour at room temperature. Following rinses in PBS, samples were stained for Sox2, Ki67 and cleaved caspase-3 at 4 °C overnight. Respective secondary antibodies and Hoechst were added for 2 hours at room temperature at a dilution of 1:1000. Immunofluorescent images were taken using a confocal and fluorescent microscope. ImageJ was used to quantify Sox2^+^Caspase-3^+^ cells or Sox2^+^Ki67^+^ cells to determine the survival and proliferation of NSC, respectively

#### Cell Secreted Metalloproteinases

To assess metalloproteinases mediated degradation of microbeads, cultured media (CM) was collected from NSC, NSC and EC, and EC confluent monolayers following 24 hours. Degradable microbeads were resuspended in PBS and CM, and incubated for 48 hours, imaging samples using brightfield microscopy every 24 hours to visualize degradation of samples.

In order to quantify cell-secreted metalloproteinases, *in situ* zymography was performed as previously described (Bowden *et al*., 2001; Chhabra *et al*., 2012). First, 2 mg/ml gelatin was dissolved in a buffer containing 61 mM NaCl and 50 mM Na_2_B_4_O_7_ (pH 9.3) and incubated at 37 °C for 1 hour. Then, 2 mg/ml fluorescein sodium salt was added, reacting for 2 hours under constant rotation in darkness. The mixture was dialyzed at room temperature for two days, then stored at 4 °C. NSC, EC, or NSC and EC were seeded on 12 mm laminin coated coverslips (10 µg/ml) at a density of 6,000 cells/slip. One day later, samples were fixed with 4% PFA and washed with PBS 3 times. A 0.5% agarose solution in fresh digestion buffer (50 mM Tris-Cl (pH = 7.4), 150 mM NaCl, 5 mM CaCl_2_) was allowed to cool to 45–50 °C, and gelatin (control) or fluorescent gelatin were added at a 1:1 ratio. Hoechst was added (1:1000) for nuclei visualization. Solution was added in a thin layer to a pre-heated glass slide, placing the fixed cells with the cell side facing down into the solution. Following gel solidification, samples were incubated in digestion buffer at 37 °C then observed under fluorescent microscope. Mean intensity fluorescence (MIF) and cell count were quantified using ImageJ, subtracting background fluorescence and normalizing MIF to cell number.

## Animals

C57Bl6 mice (Jackson Laboratories) were maintained in the Animal Research Center at Yale University on a 12-hour light/dark cycle with free access to water and food. All mice experiments were performed under a protocol approved by Institutional Animal Care Use Committee of Yale University.

## Intra-Parenchymal Stereotaxic Injections

All grafts were carried out within a 3 hour-time window of sample preparation. After anesthetizing mice with ketamine (80 mg/kg; i.p.) and xylazine (10 mg/kg; i.p.), blank beads (n = 8), free NSC and EC (n = 6), encapsulated NSC alone (n = 8) or encapsulated NSC and EC (n = 8) were injected into the striatum at the following stereotaxic coordinates relative to bregma: + 0.75 mm anterior, + 1.5 mm lateral and 2.5 mm deep, as described previously^39^. Blank microbeads, encapsulated mono-culture, free or encapsulated co-cultures were bilaterally injected using a 10 µl Hamilton microsyringe (24-gauge), in volume of 8 µl (100 beads per injection; 10,000 cells total/hemisphere) at a rate of 0.5 µl/min. The syringe was remained in place for a period of 5 min before being withdrawn slowly to prevent potential reflux of the cells/beads. The craniotomy was sealed with bone wax, and the scalp was closed with tissue adhesive. Mice were allowed to recover on a heating pad before being returned back into their home cage. Animals were euthanized after 2, 3, 7 and 14 dpt for histological analyses.

## Tissue Processing and Immunohistochemistry

Mice were perfused with 0.01 M PBS, followed by 4% PFA by cardiac puncture. Brains were removed and post-fixed overnight in 4% PFA at 4 °C prior to cryoprotection in sucrose at 4 °C for up to 48 h. Brains were cryosectioned coronally at 50 µm thickness. Sections were incubated with blocking solution (PBS containing 1% bovine serum albumin, 10% normal donkey serum and 0.1%Triton X-100) for 1 hour at room temperature then incubated overnight with primary antibodies diluted in blocking solution. The following antibodies were utilized in this study: cleaved caspase-3, CD45, doublecortin (DCX), Hoechst, Iba-1, Ki67, and Sox2. Appropriate secondary antibodies were used at a dilution of 1:500 and sections were coverslipped using Dako Fluorescence Mounting Medium. Samples were observed under a fluorescent microscope, spinning disk confocal, SP5 confocal and ophthalmology confocal microscope. Quantification of NPSC proliferation (Sox2^+^Ki67^+^) was performed manually, whereas for the NPSC quiescence and differentiation were analyzed automatically (% of area covered by respective cells) using ImageJ.

### Statistics

All statistical analyses were performed using GraphPad Prism 7 software. Unless indicated otherwise, a sample size of 3 was conducted for *in vitro* studies and a sample size of 3–8 for *in vivo* studies. Significance was determined with either a one-way ANOVA with multiple comparisons, or by an unpaired t-test as appropriate. Data are expressed as mean ± SEM and the differences were considered significant at *P* values of <0.05.

## Supplementary information

Supplemental information
