# Author Correction: Minimally Invasive Delivery of Microbeads with Encapsulated, Viable and Quiescent Neural Stem Cells to the Adult Subventricular Zone

**DOI:** 10.1038/s41598-020-57577-8

**Published:** 2020-01-14

**Authors:** Rita Matta, Seyoung Lee, Nafiisha Genet, Karen K. Hirschi, Jean-Leon Thomas, Anjelica L. Gonzalez

**Affiliations:** 1 Department of Biomedical Engineering, Yale University School of Medicine, New Haven, CT 06511 United States; 2 Department of Neurology, Yale University School of Medicine, New Haven, CT 06511 United States; 3 Yale Stem Cell Center, Yale University School of Medicine, New Haven, CT 06511 United States; 4 Yale Cardiovascular Research Center, Yale University School of Medicine, New Haven, CT 06511 United States; 5 Sorbonne Universités, UPMC Université Paris 06, Institut National de la Santé et de la Recherche Médicale U1127, Centre National de la Recherche Scientifique, AP-HP, Institut du Cerveau et de la Moelle Epinière, Hôpital Pitié-Salpêtrière, Paris, France

Correction to: *Scientific Reports* 10.1038/s41598-019-54167-1, published online 28 November 2019

This Article contains an error in Figure 2 where a duplicate of Fig. 2C (Ki67 staining) was inadvertently copied into Fig. 2D (cleaved caspase-3 staining) of NSC at day 1, during reassembly of the manuscript. The correct Figure 2 appears below as Figure 1.

**Figure 1:**
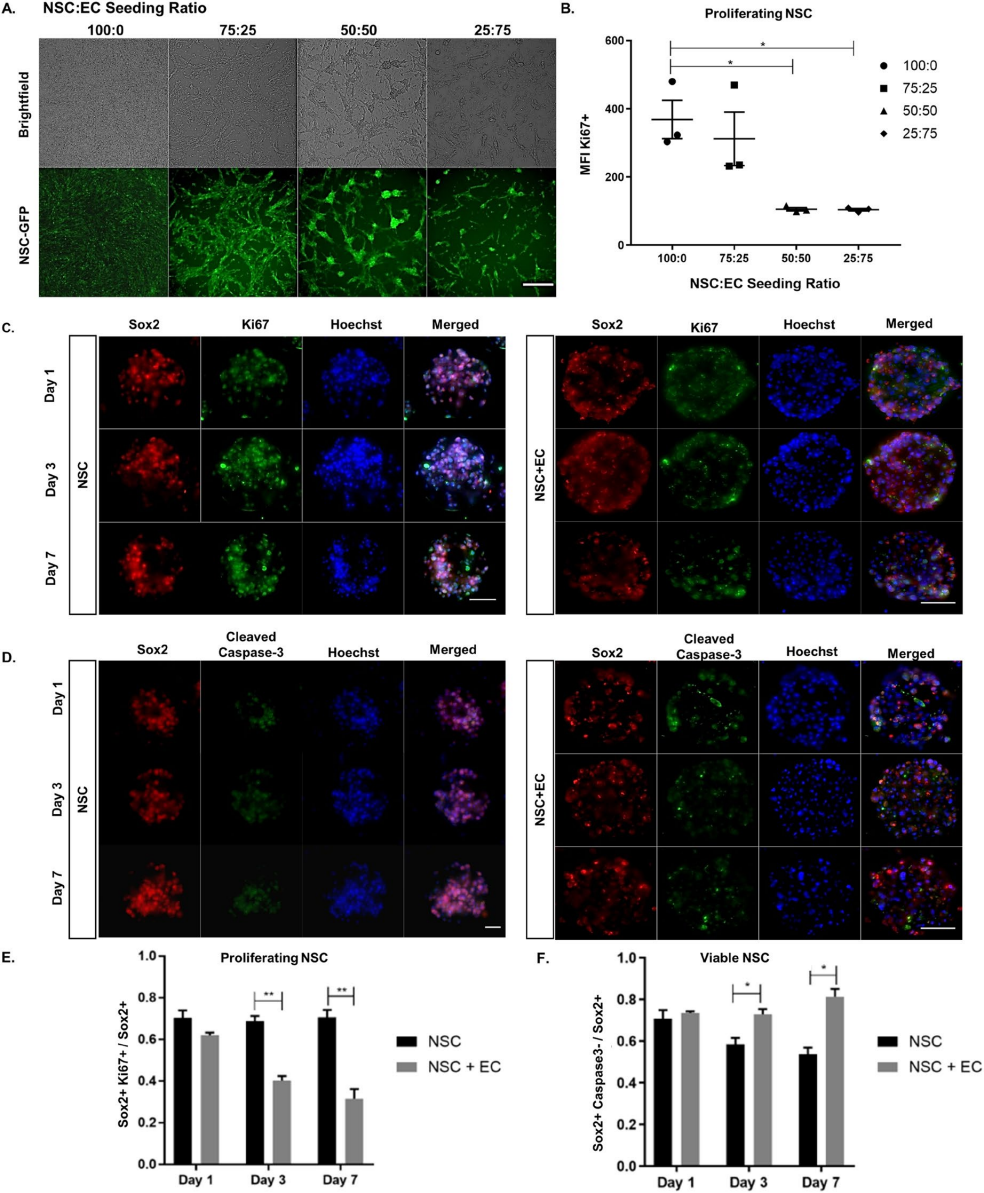
.

