# Supplementary material for: Minimally Invasive Delivery of Microbeads with Encapsulated, Viable and Quiescent Neural Stem Cells to the Adult Subventricular Zone
Source: Sci Rep. 2019 Nov 28;9:17798. doi: 10.1038/s41598-019-54167-1 (PMC6882840; doi:10.1038/s41598-019-54167-1)
Supplement: Supplementary file 1 — Supplemental information [file 41598_2019_54167_MOESM1_ESM.pdf]

Minimally Invasive Delivery of Microbeads with Encapsulated, Viable and Quiescent Neural Stem Cells to the Adult Subventricular Zone

Rita Matta*[1], Seyong Lee*[2,3], Nafiisha Genet[3,4], Karen K. Hirschi[1,3,4], Jean-Leon Thomas[2,3,5], and Anjelica L. Gonzalez[1]

[1]Department of Biomedical Engineering, [2]Department of Neurology, [3]Yale Stem Cell Center, Yale University School of Medicine, [4]Yale Cardiovascular Research Center, Yale University School of Medicine, New Haven, CT 06511, United States, [5]Sorbonne Universités, UPMC Université Paris 06, Institut National de la Santé et de la Recherche Médicale U1127, Centre National de la Recherche Scientifique, AP-HP, Institut du Cerveau et de la Moelle Epinière, Hôpital Pitié-Salpêtrière, Paris, France
Contact
Anjelica L. Gonzalez
anjelica.gonzalez@yale.edu
Jean-Leon Thomas
jean-leon.thomas@yale.edu

Supplementary Figure 1: Degradation of NSC Encapsulated Microbeads

A) As a control, NSC were plated in PEG polymer and pre-gel solution, where cells remain healthy after 72 hours. This verifies that the polymer and photocrosslinking chemicals are not toxic to the cells in culture. B) Degradable microbeads in media (first panel), CSF (second panel), and 2 mg/ml collagenase (third panel) with encapsulated NSC. Cells loosely encapsulated at the surface of microbeads adhere and elongate on the well plate as seen at 48 and 72 hours (indicated by red arrows). Microbeads swell in the nutrient rich CSF, and degrade within 24 hours in the collagenase solution.

Scale bar (100 µm) representative of all images.

Supplementary Figure 2: Astrocytic and Microglial Reaction to Needle Stab

Illustrative image of astrocytic and microglial reaction to intra striatal injection. A) Astrocytes GFAP+ were visualized around the region of needle trajectory to lateral ventricle (LV) and subventricular zone (SVZ). B) A similar pattern was observed in microglial cells $Iba1^+$ around the injection site.

Scale bar (50 µm) representative of all images.

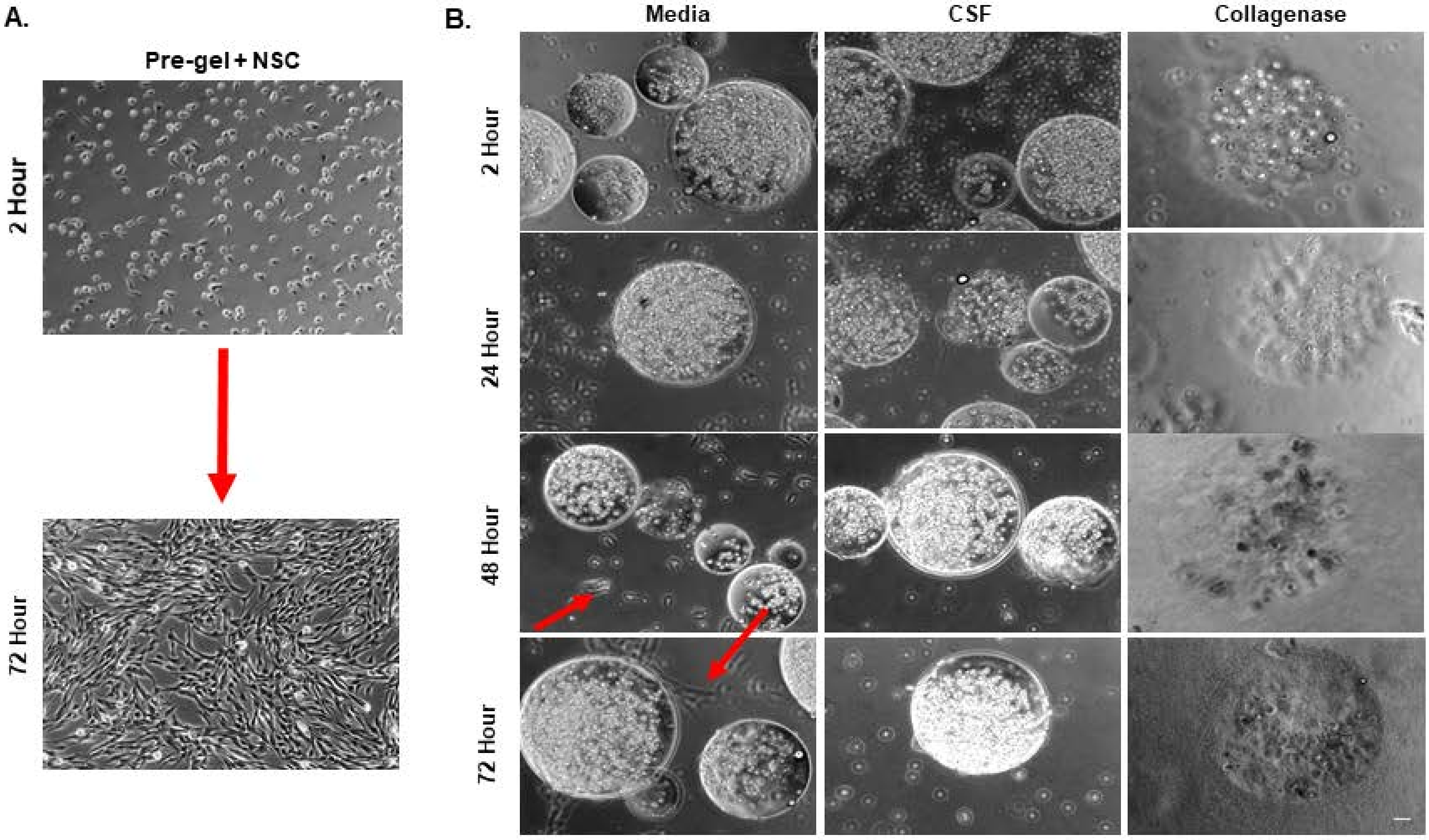
A.
Pre-gel + NSC
2 Hour
72 Hour
B.
Media
CSF
Collagenase
2 Hour
24 Hour
48 Hour
72 Hour

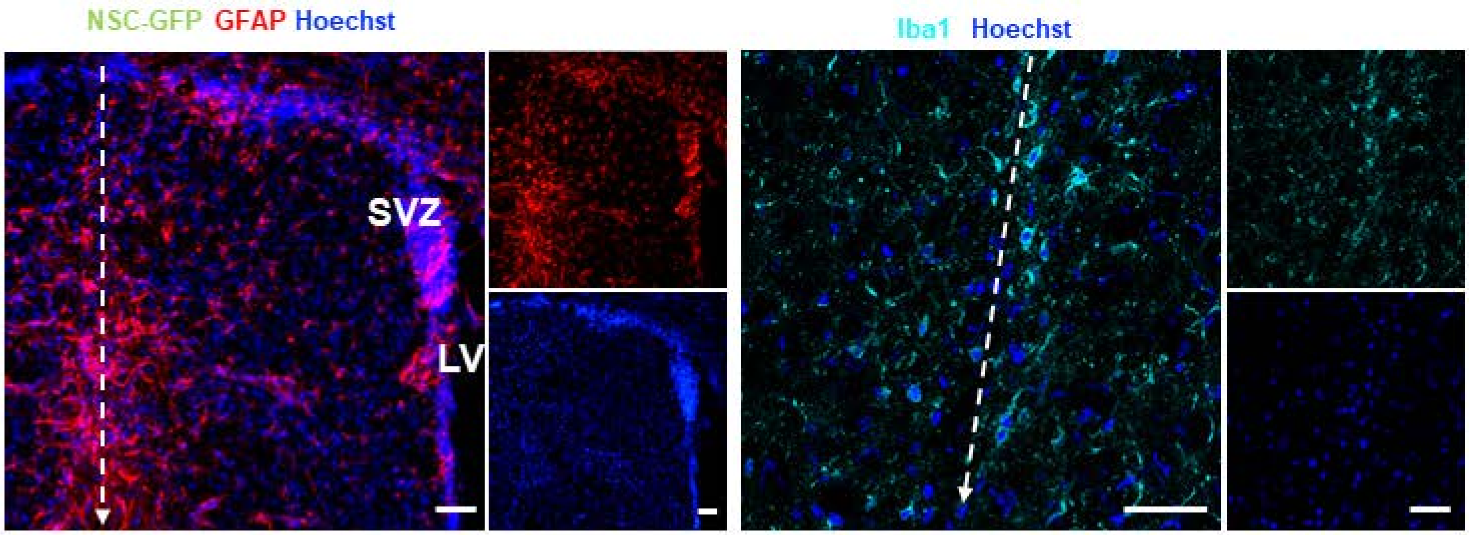
NSC-GFP GFAP Hoechst
SVZ
LV
Iba1 Hoechst
